# A study of gender differentials in the prevalence of tuberculosis based on NFHS-2 and NFHS-3 data

**DOI:** 10.4103/0970-0218.66869

**Published:** 2010-04

**Authors:** P P Sharma, Ashok Kumar, Padam Singh

**Affiliations:** LRS Institute of TB and Respiratory Diseases, (Near Qutab Minar), Sri Aurobindo Marg, New Delhi, India; 1 Department of Statistics, MD University, Rohtak, Haryana, India; 2 Member National Statistical Commission, 2^nd^ Floor, Sardar Patel Bhawan, New Delhi, India

**Keywords:** Gender gap/difference in tuberculosis, India, National Family Health Survey, prevalence of tuberculosis, tuberculosis

## Abstract

**Background:**

Worldwide, the case notification rate of tuberculosis has been reported to be higher for men than women. In India also, the prevalence of TB is higher among males as compared to females but it is important to study the trend of gender gap in the prevalence of tuberculosis over the years.

**Objective:**

To examine the trend in gender gap in the prevalence of TB over the years.

**Materials and Methods:**

The unit level data of NFHS-2 (1998-99) and NFHS-3 (2005-06) has been utilized. Gender gap in the prevalence of TB has been estimated for the two rounds of the surveys. The delta (Δ), the difference in gender gap in two surveys, has been estimated and decomposed by background characteristics such as place of residence(urban/rural), religion (Hindus/Muslims/others), caste(SC/ST/OBC/others) and standard of living(SLI) (low/medium/high) categories.

**Main Findings:**

Overall, the prevalence of TB has remained almost same in the two surveys [432/lakh in NFHS-2 and 418/lakh in NFHJS-3; *Z*=1.19, *P*=0.275. The gender gap has increased to 217/lakh in NFHS-3 in comparison to 145 per lakh in NFHS-2. The increase in gender gap is significantly higher in rural areas [of 98 per lakh;167/ lakh in NFHS-2 *vs* 265/lakh in NFHS-3; *P*<0.05] as compared to corresponding increase in urban areas [of 30 per lakh; 88/ lakh in NFHS-2 vs118/ lakh in NFHS-3, *P*>0.05]. The increase in delta (D) (difference in gender gap in two surveys) is accounted for as 88% by the rural areas and 12% by the urban areas.

**Conclusion:**

The increase in gender gap in the prevalence of TB is more in rural areas as compared to urban areas. The increase in rural areas is mainly contributed by Hindus, SC and ST and low and medium SLI categories and in urban areas, the contribution is mainly by Hindus, other castes and high SLI categories.

## Introduction

Worldwide, more men than women are known to be suffering from tuberculosis. As the tuberculosis affects the most productive age groups, the impact of the disease is felt by the children and their families. In India also, the prevalence of TB is higher among males as compared to females. However, the gap in the prevalence of TB among males and females has shown a widening trend over years which needs an in-depth examination.

Present study is an attempt to examine the difference in gender gaps in the prevalence of tuberculosis among different religions, social groups (caste), standard of living and residence categories.

## Materials and Method

For the present study, the unit level data of NFHS-2 (1998-99)(1) and NFHS-3 (2005-06)(2) has been utilized. The information in these surveys relate to more than 90,000 households.

In NFHS, the questions asked about TB were *Does any usual resident of your household suffer from tuberculosis* For each household member identified as suffering from TB; the respondent was asked *has the person suffering from TB received medical treatment for tuberculosis*. In the present study, a case of tuberculosis is defined as those who have reported to be medically treated for tuberculosis.

The information on the background characteristics of households such as religion and caste as well as residence (rural/urban) has been collected. Each household has been assigned standard of living index (SLI) code based on housing characteristics and ownership of assets. On the basis of these, each household has been classified into low, medium and high standard of living (SLI) categories. All individuals in the same household are assigned the same SLI category.

The prevalence of TB has been calculated separately for males and females respondents according to religion, caste groups (SC, ST, OBC, others) and standard of living index (low, medium and high) categories. This has been used in studying the gender gap and also its decomposition by religion, caste, SLI categories for rural and urban areas.

### Statistical analysis

The analysis was focused on the following factors:

- Calculation of gender gaps for 1998-1999 and 2005-2006.
- Computing of difference in gender gaps for 1998-99 and 2005-2006.
- Testing for the significance of the difference in gender gaps.
- Decomposing the difference in gender gaps by background characteristics.

These are explained as follows:

The gender gaps in the prevalence of TB was calculated as the difference in the prevalence of TB among males and females.

The parameter of interest was:

Delta (Δ) = Difference in gender gaps in two surveys = (Gender gap)_NFHS-3_ − (Gender gap)_NFHS-2_ = {(Prevalence of TB among males- Prevalence of TB among females)}_NFHS-3_ − }(Prevalence of TB among males- Prevalence of TB among females)}_NFHS-2_

The gender gap was calculated separately for all the studied background characteristics.

### Calculation of standard error of delta

Let P_M_ =Prevalence of TB among males

P_F_ =Prevalence of TB among females

$$\mathrm{Delta}\;\left(\Delta \right)={\left(P_{M}-P_{F}\right)}_{\mathrm{NFHS3}}\;-\;{\left(P_{M}-P_{F}\right)}_{\mathrm{NFHS2}}$$

$$\mathrm{Var}\left(\Delta \right)=\mathrm{Var}\left[{\left(P_{M}-P_{F}\right)}_{\mathrm{NFHS3}}\;-\;{\left(P_{M}-P_{F}\right)}_{\mathrm{NFHS2}}\right]$$

$$=\mathrm{Var}{\left(P_{M}-P_{F}\right)}_{\mathrm{NFHS3}}\;+\;\mathrm{Var}{\left(P_{M}-P_{F}\right)}_{\mathrm{NFHS2}}$$

$$=\mathrm{Var}{\left(P_{M}\right)}_{\mathrm{NFHS3}}+\mathrm{Var}{\left(P_{F}\right)}_{\mathrm{NFHS3}}+\mathrm{Var}{\left(P_{M}\right)}_{\mathrm{NFHS2}}+\mathrm{Var}{\left(P_{F}\right)}_{\mathrm{NFHS2}}$$

$$=\frac{\left(P_{M_{\mathrm{NFHS3}}}\right)\left(Q_{M_{\mathrm{NFHS3}}}\right)}{\left(n_{M_{\mathrm{NFHS3}}}\right)}+\frac{\left(P_{F_{\mathrm{NFHS3}}}\right)\left(Q_{F_{\mathrm{NFHS3}}}\right)}{\left(n_{F_{\mathrm{NFHS3}}}\right)}+\frac{\left(P_{M_{\mathrm{NFHS2}}}\right)\left(Q_{M_{\mathrm{NFHS2}}}\right)}{\left(n_{M_{\mathrm{NFHS2}}}\right)}+\frac{\left(P_{F_{\mathrm{NFHS2}}}\right)\left(Q_{F_{\mathrm{NFHS2}}}\right)}{\left(n_{F_{\mathrm{NFHS2}}}\right)}$$

$$\mathrm{SE}\left(\Delta \right)=\mathrm{SQRT}\;\left[\mathrm{Var}\left(\Delta \right)\right]$$

Test of significance for delta(Δ) was done by Z- score as follows:

$$Z\;=\;\left(\frac{\left(\Delta \right)}{\mathrm{SE}(\Delta )}\right)$$

Thereafter, the decomposition of delta (Δ) into its constituents was calculated.

The overall share of rural and urban areas in delta is calculated as follows:

Let

Δ_R_ =Difference in gender gap in rural areas.

Δ_U_ =Difference in gender gap in urban areas.

n _R_ =Sample size in rural areas in NFHS-3.

n _U_ =Sample size in urban areas in NFHS-3.

C _R_ =Share of rural areas in delta (%).

$$C_{R}\;=\frac{\left(n_{R}\right)\left(\Delta_{R}\right)}{\left[\left(\left(n_{R}\right)\left(\Delta_{R}\right)\right)+\left(\left(n_{U}\right)\left(\Delta_{U}\right)\right)\right]}\times 100$$

Similarly, share of urban areas in delta is given by

$$C_{U}\;=\frac{\left(n_{U}\right)\left(\Delta_{U}\right)}{\left[\left(\left(n_{R}\right)\left(\Delta_{R}\right)\right)+\left(\left(n_{U}\right)\left(\Delta_{U}\right)\right)\right]}\times 100$$

Further, these deltas (difference in gender gaps) in urban and rural areas were decomposed by background characteristics such as religion, social groups and standard of living. The same is explained for one of the characteristics say caste.

Let n_SC_ =Sample size for SC.

n_ST_ =Sample size for ST.

n_OBC_=Sample size for OBC.

n_O_=Sample size for others.

Δ_SC_ =Difference in gender gap in SC.

Δ_ST_ =Difference in gender gap in ST.

Δ_OBC_ =Difference in gender gap in OBC.

Δ_O_ =Difference in gender gap in others.

The decomposition of C_R_ by caste for SC, ST, OBC and others is given by

$$C_{\mathrm{SC}}\;=\;\frac{\left(n_{\mathrm{SC}}\right)\left(\Delta_{\mathrm{SC}}\right)}{\left[\left(n_{\mathrm{SC}}\right)\left(\Delta_{\mathrm{SC}}\right)+\left(n_{\mathrm{ST}}\right)\left(\Delta_{\mathrm{ST}}\right)+\left(n_{\mathrm{OBC}}\right)\left(\Delta_{\mathrm{OBC}}\right)+\left(n_{O}\right)\left(\Delta_{O}\right)\right]}\times C_{R}$$

$$C_{\mathrm{ST}}\;=\;\frac{\left(n_{\mathrm{ST}}\right)\left(\Delta_{\mathrm{ST}}\right)}{\left[\left(n_{\mathrm{SC}}\right)\left(\Delta_{\mathrm{SC}}\right)+\left(n_{\mathrm{ST}}\right)\left(\Delta_{\mathrm{ST}}\right)+\left(n_{\mathrm{OBC}}\right)\left(\Delta_{\mathrm{OBC}}\right)+\left(n_{O}\right)\left(\Delta_{O}\right)\right]}\times C_{R}$$

$$C_{\mathrm{OBC}}\;=\;\frac{\left(n_{\mathrm{OBC}}\right)\left(\Delta_{\mathrm{OBC}}\right)}{\left[\left(n_{\mathrm{SC}}\right)\left(\Delta_{\mathrm{SC}}\right)+\left(n_{\mathrm{ST}}\right)\left(\Delta_{\mathrm{ST}}\right)+\left(n_{\mathrm{OBC}}\right)\left(\Delta_{\mathrm{OBC}}\right)+\left(n_{O}\right)\left(\Delta_{O}\right)\right]}\times C_{R}$$

$$C_{O}\;=\;\frac{\left(n_{O}\right)\left(\Delta_{O}\right)}{\left[\left(n_{\mathrm{SC}}\right)\left(\Delta_{\mathrm{SC}}\right)+\left(n_{\mathrm{ST}}\right)\left(\Delta_{\mathrm{ST}}\right)+\left(n_{\mathrm{OBC}}\right)\left(\Delta_{\mathrm{OBC}}\right)+\left(n_{O}\right)\left(\Delta_{O}\right)\right]}\times C_{R}$$

The decomposition has been done similarly for religions and SLI categories.

This analysis helps in identifying the factors showing significant contribution in delta (Δ).

## Results and Discussions

### Sample characteristics

The details of samples covered under NFHS-2 and NFHS-3 are given in Table 1.

**Table 1:** Percentage distribution of population by sample characteristics in NFHS-2 and NFHS-3

Sample characteristics	NFHS-2			NFHS-3		
	Urban %	Rural %	Combined (U+R) %	Urban %	Rural %	Combined (U+R) %
Total HH population	130336	360764	491100	162133	359894	522027
	27.0	73.0	100.0	31.0	69.0	100.0
Sex						
Male	51.9	51.1	51.9	520	50.0	50.0
Female	48.1	48.9	48.1	48.0	50.0	50.0
Religion						
Hindu	76.5	84.8	82.6	77.8	84.1	82.2
Muslim	18.7	11.9	13.7	17.9	12.7	14.3
Others	4.8	3.4	3.8	4.3	3.2	3.5
Caste						
SC	15.9	20.6	19.3	1/0	21.0	20.0
ST	3.9	11.3	9.4	3.0	11.0	9.0
OBC	31.3	35.3	34.2	38.0	43.0	41.0
Others	49.0	32.8	37.1	42.0	25.0	30.0
Standard of living						
Low	12.7	39.6	32.5	9.0	34.0	27.0
Medium	45.9	47.9	47.4	24.0	38.0	34.0
High	41.4	12.5	20.2	67.0	28.0	40.0

NFHS-2 covered a population of 4,91,100 of which 73% belonged to the rural areas and 27% to urban areas. Both genders were approximately equally represented. While classifying the population according to the religions, it had been found that about 82.6% belonged to Hindus (including Sikhs and Jains). Muslims comprised 13.7% of population and 3.8% to all other religions. Further, 19.3% belonged to SC, 9.4% to Scheduled Tribes, 34.2% as OBC and 37.1% as other castes. On the basis of standard of living categories, 32.5% of the sample belonged to low standard of living, 47.4% to medium and 20.2% to high standard of living groups as shown in Table 1.

NFHS-3 covered a population of 5,22,027 of which 31% belonged to urban and 69% to rural areas. Further, according to the religions, 82.2% belonged to Hindus (including the Sikhs and Jain religions). 14.3 % belonged to Muslims and 3.5% to all other religions. Further 20% were from SC, 9% as ST, 41% as OBC and 30% as other caste groups. As per SLI categories, 27% belonged to low standard of living, 34% to medium and 40% to high standard of living categories.

### Gender gap

The overall prevalence of TB combined for urban and rural areas was 432 per lakh (1 lakh=100,000) in NFHS-2 and 418 per lakh in NFHS-3. This decline was not statistically significant (*χ*^2=^1.19; *P*= 0.275) as shown in Table 1a.

**Table 1a:** Prevalence of tuberculosis in NFHS-2 and NFHS-3

Area	% Population NFHS-2	Prevalence /lakh NFHS-2	% Population NFHS-3	Prevalence/lakh NFHS-3	Significance among prevalence rates in NFHS2,*vs* NFHS-3
Urban	27	307	31	307	*Z*=0.02
Rural	73	476	69	469	*Z*=0.50
Overall (urban+rural)	491,100 (100%)	432	522,027(100%)	418	*Z*=1.01

1 lakh=100,000; *Z*<1.96 Not significant; Figures in parenthesis are in percentages.

In both the NFHS surveys, the prevalence of TB among males is found to be higher than the females [Table 2]. The gender gap being 145 per lakh in NFHS-2 has increased to 217 per lakh in NFHS-3, the difference being statistically significant (*Z*=2.747, *P*<0.05).

**Table 2:** Overall difference [delta (Δ)] in gender gap In NFHS-2 and NFHS-3

	% Population NFHS-2	Prev/lakh NFHS-2	% Population NFHS-3	Prev/lakh NFHS-3	Significance among prevalence	Delta (Δ)	Significance of delta (Δ)
Overall							
M	51.3	502	50.3	526	*Z*=1.18		
F	48.7	357	49.7	309	*Z*=2.89#		
Gender gap		145		217		72	*Z*=2.747#
Urban							
M	13.8	350	16.1	364	*Z*=0.49		
F	12.8	262	15.0	246	*Z*=0.58		
Gender gap		88		118		30	*Z*=0.751
Rural							
M	37.5	558	34.2	602	*Z*=1.72		
F	35.9	391	34.7	337	*Z*=2.67#		
Gender gap		167		265		98	*Z*=3.011#

Delta (Δ)=Difference in gender gap; Total population of NFHS-2=491,100; Total population in NFHS-3=522,027;

# *P*<0.05 and significant; M=Male, F=Female; 1 lakh=100,000, Prev=Prevalence

While comparing the gender-wise prevalence of TB, it was found that the increase in the prevalence of TB among males was not statistically significant [502/lakh in NFHS-2 *vs* 526/lakh in NFHS-3, *Z*=1.18, *P*>0.05]. Whereas, among females, there was a statistically significant decline in the prevalence of TB in NFHS-3 in comparison to NFHS-2[357/lakh in NFHS-2 *vs* 309/lakh in NFHS-3, *Z*=2.89, *P*<0.05] and this decline in TB prevalence among females was observed particularly in rural areas [391/lakh in NFHS-2 *vs* 337/lakh in NFHS-3, *Z*=2.67, *P*<0.05].

### Religion-wise gender gap in the prevalence of tuberculosis

Table 3 presents the results on gender gap for religions. While comparing the gender gap in NFHS-2 and NFHS-3, it is observed that combined for urban and rural areas, there is a significant increase in gender gap among Hindus from 149 per lakh in NFHS-2 to 224 per lakh in NFHS-3 (*Z*=2.71, *P*<0.05). The increase of 23 per lakh for Muslim was not statistically significant. Further, this increase in gender gap among Hindus was statistically significant in rural areas (172 per lakh in NFHS-2 *vs* 266 per lakh in NFHS-3 *Z*=2.757, *P*<0.05) and not in urban areas (*Z*=1.06, *P*>0.05).

**Table 3:** Religion-wise difference [delta (Δ)] in gender gaps

Religions and sex	NFHS_2		NFHS-3		Gender gap in (NFHS_2) D1=	Gender gap in NFHS-3 D2=	Delta (Δ) =D2-D1	Significance of delta (Δ)
	% population	Prev per lakh	% population	Prev per lakh				
Urban								
Hindu								
M	10.55	316	12.58	350				
F	9.74	231	11.58	217	85	133	48	*Z*=1.06
Muslim								
M	2.55	488	2.86	442				
F	2.40	389	2.71	386	99	56	-43	*Z*=0.380
Others								
M	0.65	360	0.65	288				
F	0.64	257	0.67	169	103	119	16	*Z*=0.031
Rural								
Hindu								
M	31.87	545	28.88	582				
F	30.36	373	29.11	316	172	266	94	*Z*=2.757#
Muslim								
M	4.41	650	4.24	689				
F	4.30	478	4.50	441	172	248	76	*Z*=0.744
Others								
M	1.23	593	1.07	805				
F	1.23	517	1.13	470	76	335	259	*Z*=1.240
Urban+rural								
Hindu								
M	42.42	488	41.46	512				
F	40.09	339	40.69	288	149	224	75	*Z*=2.706#
Muslim								
M	6.96	591	7.11	589				
F	6.69	446	7.21	421	145	168	23	*Z*=0.313
Others								
M	1.88	513	1.71	610				
F	1.87	428	1.80	357	85	253	168	*Z*=1.18

1 lakh=100,000, M=Male, F=Female; Hindu=(Hindu + Sikh=Jain), Others=(Other religions + Christian);

# *P*<0.05 Significant; Delta (Δ)=Difference in gender gap; Total population of NFHS-2=491,100;Total population in NFHS-3=522,027; M=Male, F=Female; 1 lakh=100,000; *P*<0.05 and Significant

### Caste-wise (Social groups) gender gap in prevalence of tuberculosis

Table 4 presents results on gender gap for caste groups. Among the total population (combined for urban and rural) it is observed that there is a statistically significant increase in gender gap in NFHS-3 in comparison to NFHS-2 among Scheduled Tribes [181/lakh in NFHS-2 to 504/lakh in NFHS-3; *Z*=3.223, *P*<0.05] and other caste groups [56/lakh in NFHS-2 to 176/lakh in NFHS-3; *Z*=2.953, *P*<0.05]. However, the increase in gender gap is not statistically significant among the Scheduled Castes [202/lakh in NFHS-2 to 314/lakh in NFHS-3; *Z*=1.69, *P*>0.05] and OBC, though in reverse direction.

While, analyzing the data for rural areas, it is found that the gender gap was significantly higher in NFHS-3 in comparison to NFHS-2 among Scheduled Castes [202/lakh in NFHS-2 to 367/lakh in NFHS-3; *Z*=2.113, *P*<0.05]; Scheduled Tribes [208 /lakh in NFHS-2 to 517/lakh in NFHS-3; *Z*=2.88, *P*<0.05] and other castes [93/lakh in NFHS-2 to 249/lakh in NFHS-3; *Z*=2.792, *P*<0.05].

However, no significant increase was observed in urban areas for any of these castes.

**Table 4:** Caste-wise difference [delta (Δ)] in gender gap

Caste and sex	NFHS-2		NFHS-3		Gender gap in (NFHS-2) D1=	Gender gap in NFHS-3 D2=	Delta (Δ)=D2-D1	Significance of delta (Δ)
	% population out of 491100	Prev per lakh	% population out of 522027	Prev per lakh				
Urban								
SC								
M	2.05	544	2.69	557				
F	1.89	338	2.53	379	206	178	-28	*Z*=0.286
ST								
M	0.49	290	0.45	636				
F	0.47	335	0.42	232	-45	404	449	*Z*=1.834
OBC								
M	4.01	419	5.97	332				
F	3.74	223	5.60	231	196	101	-95	*Z*=1 333
Others								
M	6.31	255	6.62	301				
F	5.82	268	6.08	214	-13	87	100	*Z*=1.758
Rural								
SC								
M	7.35	644	6.91	727				
F	6.97	442	6.95	360	202	367	165	*Z*=2.113 #
ST								
M	3.99	645	3.78	875				
F	3.89	437	3.82	358	208	517	309	*Z*=2.879 #
OBC								
M	12.53	591	14.11	499				
F	12.06	389	14.37	363	202	136	-66	*Z*=1.225
Others								
M	11.73	436	8.26	501				
F	11.14	343	8.41	252	93	249	156	*Z*=2.792 #
Urban+rural								
SC								
M	9.40	622	9.59	679				
F	8.86	420	9.48	365	202	314	112	*Z*=1.691
ST								
M	4.48	607	4.23	850				
F	4.35	426	4.24	346	181	504	323	*Z*=3.226 #
OBC								
M	16.54	549	20.07	449				
F	15.80	349	19.96	326	200	123	-77	*Z*=1.779
Others								
M	18.04	373	14.89	412				
F	16.97	317	14.49	236	56	176	120	*Z*=2.953 #

# *P*<0.05 Significant;

M=Male, F=Female; 1 lakh=100,000; Delta (Δ)=Difference in gender gap; Total population of NFHS-2=491,100; Total population in NFHS-3=522,027

### Standard of living (SLI) category-wise gender gap in prevalence of tuberculosis

Table 5 presents the results on gender gap for standard of living. It has been found among the total population (combined for urban and rural) that there is a statistically significant increase in gender gap in the prevalence of tuberculosis in NFHS-3 in comparison to NFHS-2 among all the SLI categories, for low SLI [276/lakh in NFHS-2 to 409/lakh in NFHS-3; *Z*=2.732, *P*<0.05], for medium SLI [121/lakh in NFHS-2 to 224/lakh in NFHS-3; *Z*=2.486, *P*<0.05] and for high SLI[-9/lakh(opposite direction) in NFHS-2 to 99/lakh in NFHS-3; *Z*=3.04, *P*<0.05].

**Table 5:** Standard of living (SLI) category-wise difference [delta (Δ)] in gender gap

SLI and sex	% population in NFHS-2 out of 491100	Prev/lakh	% population in NFHS-3 out of 522027	Prev/lakh	Gender gap in NFHS-2 D1=	Gender gap in NFHS-3 D2=	Delta(Δ) D2-D1	Significance of delta (Δ)
Urban							
Low								
M	1.70	799	1.41	760				
F	1.62	474	1.43	363	325	397	72	*Z*=0.447
Medium								
M	6.26	409	3.79	419				
F	5.72	295	3.49	328	114	91	-23	*Z*=0.301
High								
M	5.57	147	10.48	291				
F	5.25	163	9.73	190	-16	101	117	*Z*=2.607#
Rural								
Low								
M	14.55	750	11.38	878				
F	14.23	480	11.99	467	270	411	141	*Z*=2.25#
Medium								
M	17.92	482	12.87	588				
F	16.87	357	12.88	324	125	264	139	*Z*=2.88#
High								
M	4.66	249	9.36	287				
F	4.42	251	9.31	190	-2	97	99	*Z*=1.789
Urban+Rural								
Low								
M	16.25	755	12.79	865				
F	15.85	479	13.42	456	276	409	133	*Z*=2.73#
Medium								
M	24.18	463	16.67	549				
F	22.59	342	16.37	325	121	224	103	*Z*=2.486#
High								
M	10.24	194	19.85	289				
F	9.67	203	19.04	190	-9	99	108	*Z*=3.04#

1 lakh=100,000, SLI=Standard of living index; M=Male, F=Female; Delta (Δ)=Difference in gender gap; Total population of NFHS-2=491,100; Total population in NFHS- 3=522,027

# *P*<0.05 Significant;

While analyzing the data for rural and urban areas separately, it is found that in rural areas the gender gap has increased significantly in NFHS-3 in comparison to NFHS-2 in low SLI [270/lakh in NFHS-2 to 411/lakh in NFHS-3; *Z*=2.25, *P*<0.05] and medium SLI [125/lakh in NFHS-2 to 264/lakh in NFHS-3; *Z*=2.88, *P*<0.05], but not significant in high SLI[-2/lakh (opposite direction) in NFHS-2 to 97/lakh in NFHS-3; *Z*=1.79, *P*>0.05].

Surprisingly in urban areas, among high SLI groups, the gender gap is found to have increased significantly in NFHS-3 in comparison to NFHS-2 [-16/lakh (opposite direction) in NFHS-2 to 101/lakh in NFHS-3; *Z*=2.61, *P*<0.05]. The increase was not significant in low SLI [325/lakh in NFHS-2 to 397/lakh in NFHS-3; *Z*=0.447, *P*>0.05] and in medium SLI category (in the opposite direction).

In NFHS-3, the gender gap was significantly higher in rural areas than the urban areas only for medium SLI [91/lakh in urban *vs* 264/lakh in rural; *Z*=2.389, *P*<0.05].

### Difference (Delta (Δ)) in gender gaps in NFHS-3 vs NFHS-2

#### Overall decomposition of delta (Δ)

Overall, an increase of 72 per lakh has been observed in the increase in gender gap in NFHS-3 over NFHS-2.

Although an increase in gender gap has been observed in both rural and urban areas, it is higher in rural areas (98 per lakh) [167 per lakh in NFHS-2 and 265 per lakh in NFHS-3] as compared to corresponding increase of 30 in urban areas [88 per lakh in NFHS-2 against 118 per lakh in NFHS-3]. It may be mentioned that the delta (Δ) in gender gap is three times higher in rural areas than in urban areas.

On the basis of the calculations shown in the section ‘Materials and Method’, this difference Δ is accounted for as 88% by the rural areas and 12% by the urban areas [Table 6a].

**Table 6a:** Difference [delta (Δ)] in gender gap in the prevalence of TB and contribution in [delta (Δ)] by place of residence

Place of residence	Gender gap/lakh in NFHS_2	Gender gap/ lakh in NFHS-3	Delta (Δ)$	(%) Population NFHS-3#	Contribution in delta (%)*
Overall	145	217	72		
Urban	88	118	30	31%	12%
Rural	167	265	98	69%	88%
Urban+rural				100%	100%

$ Delta (Δ)=Difference in gender gap in NFHS-3 and NFHS-2.

# Total population in NFHS-3=522,027;

* Calculations done as per formula in materials and method;

1 lakh=100,000

### Contribution of background characteristics in delta (Δ)

Of the total 88% contribution of delta by rural areas, it is observed that its decomposition by religion is 72% by Hindus, 9% by Muslims and 8% by others. The decomposition by caste is SC 38%, ST 39%, others 43% and in opposite direction OBC as 31%. By standard of living (SLI) categories, the decomposition is low SLI 33%, medium SLI 36% and high SLI 19% as shown in Table 6b.

**Table 6b:** Difference in gender gap [delta (Δ)] in the prevalence of TB and contribution in [delta (Δ)] by background characteristics

	Delta (Δ) urban$	Per cent population urban NFHS-3#	Contribution in delta (%)* urban	Delta (Δ) rural$	Per cent population rural NFHS-3# (%)	Contribution in delta (%)* rural	Delta (Δ) urban+rural$	Per cent population of total NFHS-3 # (%)	Contribution in delta (%)* urban +rural
Hindu	48	77.9	14.8	94	84.0	71.8	75	82.1	87.0
Muslim	-43	17.9	-3.1	76	12.7	8.8	23	14.3	4.6
Others	16	4.2	0.3	259	3.2	7.5	168	3.5	8.3
			12.0			88.0			100.0
Caste									
SC	-28	16.8	-4.2	165	20.1	37.6	112	19.1	40.2
ST	449	2.8	11.2	309	11.0	38.6	323	8.5	51.5
OBC	-95	37.3	-31.8	-66	41.3	-30.9	-77	40.0	-58.0
Others	100	41.0	36.7	156	24.2	42.7	120	29.4	66.3
			12.0			88.0			100.0
Low	72	9.2	1.0	141	33.9	33.3	133	26.2	31.4
Medium	-23	23.5	-0.8	139	37.3	36.1	103	33.0	30.7
High	117	65.2	11.8	99	27.1	18.7	108	38.9	37.9
			12.0			88.0			100.0

$ Delta (Δ)=Difference in gender gaps in NFHS-3 and NFHS-2;

# urban population =31% of 522027; Rural population =69% of 522027;

* Calculations are based on numbers and not on percentages as per formula

Similarly, of the 12% share of urban areas, It is observed that its decomposition by religion is Hindus 15% and Muslims 3% in opposite direction. The decomposition by caste is ST 11%, other castes 37% and in opposite direction SC 4% and OBC 32%. The decomposition by standard of living (SLI) categories indicates that the contribution is by high SLI only.

## Discussion

Many studies(3–6) have reported the gender difference in tuberculosis, but analyzing the difference in gender gap in two consecutive national NFHS surveys and its contribution according to place of residence, religion, caste and standard of living has been done in the present study only. To our knowledge, this is the only study in which the two Indian national surveys have been studied for gender gap in the prevalence of tuberculosis.

The serious question is why there is an increasing trend in the gender gap which has been observed in the two surveys.

Many reasons can be speculated for this widening trend in the gender gap. Few of them are given below:

- Some worldwide studies(7,8) have shown that males are having higher risk factors like smoking, alcoholism and drug addiction to get tuberculosis than females. In one of the study from Hong Kong, Leung *et al*.(7) has mentioned that smoking accounted for 32.8% [95%CI,14.9-48.0%], 8.6% [95%CI,3.3-15.1%] and 18.7% [95CI,7.7-30.4%] of the TB risk among males, females and the entire cohort, respectively. In comparison to never- smokers, current smokers had an excess risk of pulmonary tuberculosis TB [adjusted HR 2.87,95%CI,2.00-4.11,*P*<0.001].
- Also, during this period, it has also been found that there is an increase in the human immunodeficiency virus infection cases and HIV infection is found to more prevalent among the male population as compared to females.

In one of the study by Silveira *et al*.(9) in a sample of 204 HIV diagnosed cases, tuberculosis prevalence was reported to be 27%. It was also mentioned that the variables which were found to be potential risk factors were being of the male gender [odds ratio 2.49, confidence interval 1.15-5.39] and using illicit drugs [odds ratio 2.1, 95% confidence interval 1.02-4.31].

Another probable reason could be that there is higher under reporting by females because of social issues and stigma, as in the strategy of DOTS treatment, the patient has to visit the DOTS centers on alternate days for taking supervised treatment. The unmarried girls and women want the diagnosis of the TB to be kept confidential. In one of the studies, Ahsan *et al*.(10) observed that 55% of cases wanted the diagnosis of TB to be kept confidential to avoid being labeled as TB patients. A total of 85.6% of female TB patients had problems in their relationship with their spouse and family members after being diagnosed with TB. There is also a lack of health seeking behavior among women.

## Conclusion

The present study mainly examines an increasing trend in gender gap over a period of five years from NFHS-2(1998-1999) to NFHS-3(2005-2006).

Although the overall prevalence of TB has remained the same in the two survey periods, the gender gap in the prevalence of TB has widened. Particularly, the prevalence of TB among females has shown a significant decline.

Studying the delta (Δ) in gender gap, it is noted that the increase in gender gap is more in rural areas than the urban areas. Of the total increase, 88% is accounted by rural areas and 12% by urban areas. Further, the increase in rural areas is contributed by Hindus, SC and ST castes, low and medium SLI categories. In urban areas, the contribution is mainly from Hindus, other caste groups and high SLI categories.

The study requires an in-depth examination using other sources of data including service statistics on treatment seeking behavior under RNTCP for the corresponding years.
